# Roles of Human Endogenous Retroviruses and Endogenous Virus-Like Elements in Cancer Development and Innate Immunity

**DOI:** 10.3390/biom13121706

**Published:** 2023-11-24

**Authors:** Hirokazu Katoh, Tomoyuki Honda

**Affiliations:** 1 Department of Virology, Faculty of Medicine, Dentistry and Pharmaceutical Sciences, Okayama University, Okayama 700-8558, Japan; pe3w6we8@okayama-u.ac.jp; 2 Department of Virology, Okayama University Graduate School of Medicine, Dentistry and Pharmaceutical Sciences, Okayama 700-8558, Japan

**Keywords:** HERVs, LINEs, cancer, innate immunity, promoter, enhancer, interferon signaling

## Abstract

Human endogenous retroviruses (HERVs) are remnants of ancient retroviral infections in the host genome. Although mutations and silencing mechanisms impair their original role in viral replication, HERVs are believed to play roles in various biological processes. Long interspersed nuclear elements (LINEs) are non-LTR retrotransposons that have a lifecycle resembling that of retroviruses. Although LINE expression is typically silenced in somatic cells, it also contributes to various biological processes. The aberrant expression of HERVs and LINEs is closely associated with the development of cancer and/or immunological diseases, suggesting that they are integrated into various pathways related to the diseases. HERVs/LINEs control gene expression depending on the context as promoter/enhancer elements. Some RNAs and proteins derived from HERVs/LINEs have oncogenic potential, whereas others stimulate innate immunity. Non-retroviral endogenous viral elements (nrEVEs) are a novel type of virus-like element in the genome. nrEVEs may also be involved in host immunity. This article provides a current understanding of how these elements impact cellular physiology in cancer development and innate immunity, and provides perspectives for future studies.

## 1. Introduction

The human genome contains sequences derived from exogenous viruses, such as human endogenous retroviruses (HERVs), and those that behave like viruses, such as long interspersed nuclear elements (LINEs) and non-retroviral endogenous viral elements (nrEVEs) [1,2,3]. Although gene-coding sequences comprise approximately 1.5% of the human genome, one-third of the genome comprises HERVs and LINEs [4]. Accumulating evidence suggests that these sequences play roles in various biological processes such as human placental morphogenesis, the maintenance of human stem cell identity, the regulation of telomere maintenance, and immune response [5,6,7,8,9,10].

**Figure 1:**
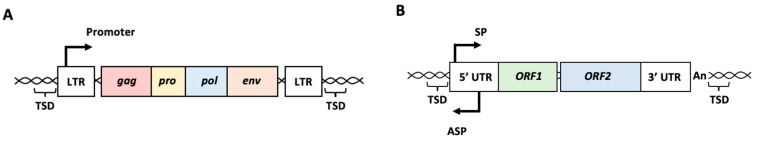
Schematic representation of intact human endogenous retrovirus (HERV) and long interspersed nuclear element-1 (LINE-1 or L1). (**A**) Intact HERV structure. Four ORFs, *gag*, *pro*, *pol*, and *env*, are surrounded with two LTRs and TSDs. Promoter activity is present in the LTR. (**B**) Intact L1 structure. Two ORFs, *ORF1* and *ORF2*, are surrounded with 5′ UTR and 3′ UTR followed by poly(A) tail. Sense and antisense promoters are present in the 5′ UTR. LTR: long terminal repeat, TSD: target site duplication, UTR: untranslated region, SP: sense promoter, ASP: antisense promoter, An: poly(A) tail.

HERVs are thought to be a legacy of historical infections caused by exogenous retroviruses [11,12]. After infection with exogenous retroviruses, the viral RNA genome is reverse-transcribed to double-stranded viral cDNA, and the viral cDNA is integrated into the host genome, forming a provirus with long terminal repeats (LTRs) on both sides [12]. Four to six bp target site duplications (TSDs) are formed during integration of the viral cDNA [12]. LTRs contain binding sites for various cellular transcription factors and polyadenylation signals. Progenic viral genomic RNA and viral mRNA are transcribed from the LTRs of the integrated proviruses. The intact provirus contains four open reading frames (ORFs) (Figure 1A). *Gag* encodes core proteins, *pro* encodes a protease for processing viral polyproteins, *pol* encodes the reverse transcriptase and integrase, and *env* encodes envelope proteins. If a germline cell is infected with a retrovirus, the integrated provirus is inherited by the offspring as Mendelian genetic elements, called HERVs. During their long-term persistence in the human genome, many mutations accumulate in HERV sequences, and most HERVs lose their coding potential. Consequently, the production of infectious particles from HERVs has not yet been demonstrated in humans [13]. However, HERVs continue to contribute to the human transcriptome, and a growing number of studies have suggested that their expression or expression products may play roles in various biological processes [5,6,7,8,9,10].

LINEs are non-LTR retrotransposons in the genome. Among the LINE family members, LINE-1 (L1) can move within the genome via a copy-and-paste mechanism, in which L1 RNA is transcribed from L1 sequences in the genome and reverse-transcribed to cDNA at new genomic sites [1,5]. Thus, the life cycle of L1 resembles that of retroviruses because it involves a reverse transcription step. A full-length L1 is approximately 6 kb in length and comprises a 5′ untranslated region (UTR), two ORFs encoding ORF1p and ORF2p, and a 3′ UTR followed by a poly(A) tail (Figure 1B) [14,15,16,17]. Typically, L1 is flanked by short stretches (between 6 and 20 bp) of identical host DNA, called TSDs. ORF1p interacts with and encapsidates L1 RNA. ORF2p possesses endonuclease and reverse transcriptase activities critical for L1 amplification. The 5′ UTR provides two internal promoters: sense and antisense. The L1 mRNA transcribed from the sense promoter is transported to the cytoplasm and binds to ORF1p and ORF2p, forming the L1 ribonucleoprotein (RNP). After the L1 RNPs are re-imported into the nucleus, the ORF2p endonuclease introduces a nick in the genomic DNA to expose the consensus oligo(dT). L1 ORF2p reverse transcriptase uses the exposed 3′ end of the oligo(dT) to prime reverse transcription. This mechanism is termed target-primed reverse transcription (TPRT). Because of TPRT, the resulting L1 cDNA is inserted into a novel locus in the genome. Because most L1s are 5′ truncated, these elements are defective; however, 80–100 copies are still retrotransposition-competent [18,19].

The mobilization of HERVs and LINEs is a threat to genome integrity because they are potentially linked to target gene disruption, alternative splicing, various epigenetic changes leading to alterations in gene expression and chromatin state, and non-allelic homologous recombination [14,15,20,21]. Therefore, the expression of HERVs and LINEs is usually repressed by epigenetic mechanisms such as DNA methylation and histone modification [13,22,23]. However, some HERVs and LINEs are still expressed and play important roles in various biological processes. For example, syncytin-1, a protein of the HERV-W family, is involved in syncytial formation [8]. Syncytin-2, a protein of the HERV-FRD family participates in syncytial formation and immune tolerance [24]. HERV-H expression is essential for maintaining human stem cell identity [9]. Furthermore, cumulative evidence shows the potential roles of HERVs in the development of diseases such as cancer and immune disorders [25]. L1 retrotransposition plays an important role in the onset of several neurological and oncological diseases in humans [26]. HERV and LINE expression can be regulated by host factors such as epigenetic modifications and external chemical or physical substances such as exogenous viral infection and environmental stresses [25,26,27].

In this review, we summarize the current understanding of the roles of HERVs and LINEs in cancer development and immunity. Several HERVs and LINEs modulate these biological processes in multiple steps. Given that the regulation of HERV and LINE expression is sensitive to external stimuli [27], an intervention that affects HERV and/or LINE expression may be a candidate for the treatment of cancers and autoimmune diseases.

## 2. Involvement of HERVs and LINEs in Cancers

Several studies have investigated the correlation between dysregulated HERV expression and various cancers including melanoma, breast cancer, urothelial carcinoma, and ovarian cancer [23,25,28,29]. The upregulation of L1s is also frequently correlated with cancer onset and progression [30]. However, causative evidence for the oncogenic potential of HERVs and L1s remains elusive [30,31]. In this section, we describe the functions of HERVs and L1s in cancer development. As DNA elements, HERVs/LTRs provide promoter/enhancer elements for nearby and/or distal gene expression, and L1 sequence insertion into or near an oncogene or tumor suppressor gene disrupts the gene-coding sequence or modulates the expression of the target gene [6,13,25,32,33]. Alternatively, HERVs regulate the expression of oncogenes or tumor suppressor genes as RNAs or proteins [25,28]. L1 chimeric transcripts or proteins can change cellular properties and affect certain biological processes, such as cell growth and metabolic processes [15,34,35,36,37,38,39].

### 2.1. HERVs and Cancers

#### 2.1.1. LTRs as Promoter/Enhancer Elements

**Figure 2:**
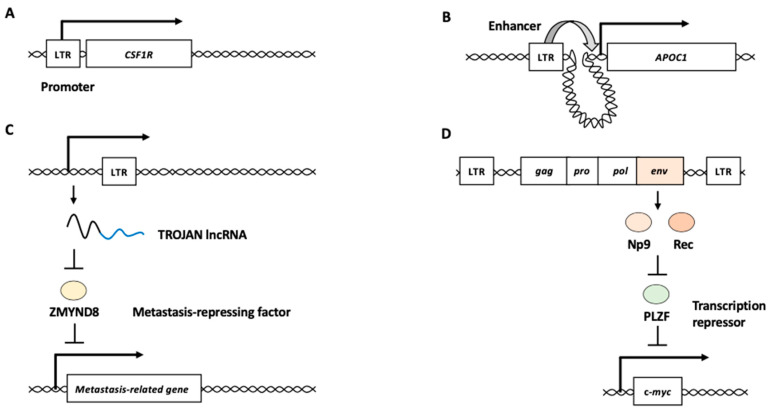
HERV functions in cancer development. (**A**) HERV LTR functions as a promoter of a nearby gene. *CSF1R* encodes the receptor for colony-stimulating factor 1 with oncogenic potential. (**B**) HERV LTR functions as an enhancer of distant gene from the element. *APOC1* is one of the targets for enhancer activity of the LTR in acute myeloid leukemia. (**C**) HERV long noncoding RNA (lncRNA) functions as an oncogenic RNA. The LTR sequence-containing lncRNA, TROJAN, is a negative regulator of ZMYND8, which is a metastasis-repressing factor. (**D**) HERV proteins function as oncogenic proteins. HERV Env proteins, Np9 and Rec, repress transcription repressor PLZF, which represses the expression of an oncogene c-*myc*. LTR: long terminal repeat.

LTRs can act as promoter/enhancer elements to modulate the expression of surrounding genes. For example, the malignant Hodgkin/Reed–Sternberg (HRS) cells of Hodgkin’s B-cell lymphoma aberrantly express the transcript of *CSF1R*, a receptor for colony-stimulating factor 1, from an LTR (THE1B) located 6.2 kb upstream of the normal myeloid transcription start site [40,41]. The prominent demethylation of CpG elements was found in the LTR region upstream *CSF1R* in several of Hodgkin’s lymphoma cell lines, suggesting that epigenetic alterations activate the LTR promoter activity for aberrant *CSF1R* expression (Figure 2A) [41]. Six ERV families (LTR2B, LTR2C, LTR5B, LTR5_Hs, LTR12C, and LTR13A) with acute myeloid leukemia (AML)-associated enhancer chromatin signatures are enriched in the binding sites of key regulators of hematopoiesis and AML pathogenesis [42]. An LTR2 is located upstream of the apolipoprotein C1 (*APOC1*) promoter and acts as an enhancer (Figure 2B) [42]. The genetic or epigenetic perturbation of the LTR2 leads to the reduced expression of *APOC1*, restricting cellular proliferation and increasing apoptosis in malignant cells [42]. These studies demonstrate that HERV LTRs participate in human carcinogenesis by modifying the expression of host genes as promoter/enhancer elements.

#### 2.1.2. HERV-Derived RNAs and Proteins

Several studies have suggested potential roles of HERV-derived RNAs and proteins in cancer development. For example, a genome-wide transcriptome analysis of HERVs revealed that a long noncoding RNA (lncRNA), named TROJAN, is highly expressed in triple-negative breast cancer (TNBC) [43,44]. The 3′ end of TROJAN contains several mosaic LTRs. TROJAN binds to ZMYND8, a metastasis-repressing factor, and increases ZMYND8 degradation through the ubiquitin–proteasome pathway (Figure 2C) [45]. Metastasis-related genes such as *EGFR*, *VEGFA*, and *MDM2* are directly regulated by both TROJAN and ZMYND8 [44]. Antisense oligonucleotide therapy targeting TROJAN substantially suppresses TNBC progression in vivo [44]. These findings suggested that TROJAN serves as an oncogenic lncRNA in TNBC.

HERV-K is the most recently inserted HERV subfamily, comprising 30–50 proviruses in the human genome [46,47]. The expression levels of HERV-K *env* mRNA are increased in hepatocellular carcinoma (HCC) and are associated with cancer progression and poor outcomes [48]. The in vitro growth rates of pancreatic cancer cell lines were reduced after HERV-K *env* knockdown, and the knockdown cells exhibited reduced lung metastasis [49]. HERV-K *env* transcripts produce two oncogenic proteins, Np9 and Rec, that modulate cellular gene expression and induce cancer development [24,50]. The *np9* mRNA is expressed in various tumor tissues and transformed cell lines, but not in normal non-transformed cells [51]. Both Np9 and Rec interact with the promyelocytic leukemia zinc finger (PLZF) tumor suppressor, a transcriptional repressor and chromatin remodeler implicated in cancer [52]. One of the major targets of PLZF is the c-*myc* oncogene [52]. Np9 and Rec may exhibit oncogenic potential by derepressing c-*myc* through PLZF inhibition (Figure 2D). Furthermore, Np9 can activate β-catenin, extracellular signal–regulated kinase (ERK), Akt, and Notch1 signaling pathways and promote the growth of human myeloid and lymphoblastic leukemia cells [53]. The expression of HERV-K *pol* mRNA in bone marrow mononuclear cells is higher in patients with leukemia than in healthy donors [54].

More than 200 HERV-W elements have been identified in the human genome [55]. Syncytin-1 is an envelope protein of HERV-W. Syncytin-1 is specifically expressed in the human placenta and mediates trophoblast cell fusion in multinucleated syncytiotrophoblast layers [56]. Syncytin-1 is involved in several types of cancers [24,57]. Syncytin-1 is upregulated in endometrial carcinoma, and the overexpression of syncytin-1 can promote cell proliferation, cell cycle progression, and invasion of endometrial carcinoma cells [58,59]. Syncytin-1 expression is upregulated in HCC and enhances cell proliferation, metastasis, and tumorigenicity [60]. Syncytin-1 promotes HCC cell proliferation via the MEK/ERK pathway [60]. Syncytin-1 overexpression increased the proliferation and viability of immortalized human uroepithelial cells, suggesting its participation in uroepithelial cell carcinoma tumorigenesis [61].

HERV-H is an abundant HERV subfamily in the human genome, with more than 1000 copies, including full-length and truncated forms, and solitary LTRs [62,63]. HERV-H is highly expressed in both naïve and primed stem cells and is essential for pluripotency [9]. A HERV-H copy located on Xp22.3, encompassing a potential ORF immediately downstream of the LTR, was overexpressed in 16 of 34 (47%) colorectal, 25 of 63 (40%) gastric, and two of 12 (17%) pancreatic cancers [64]. RNA from an X-linked member of the HERV-H family is frequently expressed in colon cancer, but not in normal tissues [65].

These examples demonstrate that HERV-derived RNAs and proteins play essential roles in cancer development by modulating transcriptional regulation and downstream signaling.

### 2.2. L1 and Cancers

#### 2.2.1. L1 Insertional Mutagenesis

**Figure 3:**
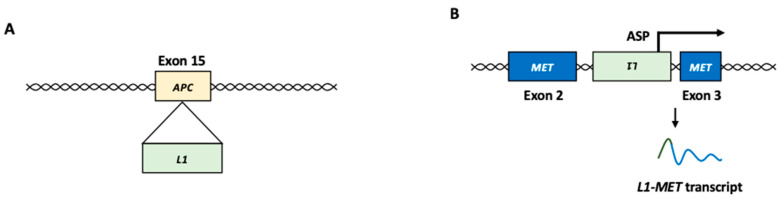
L1 function in cancer development. (**A**) Insertional mutagenesis. In colon cancer, L1 insertion into exon 15 of the *APC* gene, which encodes a tumor suppressor, resulted in disruption of the gene function. (**B**) *L1-MET* transcript. L1 antisense promoter can produce L1 chimeric transcripts fused to a nearby gene, such as *MET* oncogene. L1: LINE-1, ASP: antisense promoter.

The insertion of L1 within or near oncogenes or tumor suppressor genes can contribute to tumor development [6,14,15,32,66,67]. For example, *telomerase reverse transcriptase* (*TERT*) is one of the most commonly associated genes with L1 insertions [68,69]. Since aberrant TERT expression is associated with tumor development, L1 insertion near the *TERT* locus may play a role in carcinogenesis [70,71]. The somatic insertion of the L1 element was identified in exons 15 and 16 of the *APC* gene in colon and colorectal cancer, respectively (Figure 3A) [72,73]. The disruption of the *APC* gene, a tumor suppressor, can play a role in the constitutive activation of the Wnt/β-catenin pathway [74]. Germline L1 insertion in the *mutated in colorectal cancers* (*MCC*) gene was observed in the genomes of patients with HCC [75]. MCC is a β-catenin-interacting protein that can act as a potential tumor suppressor by inhibiting the Wnt/β-catenin pathway [76]. In some cases, the L1 insertion upregulates the expression of target genes. L1 insertion in the *suppression of tumorigenicity 18* (*ST18*) gene, a transcriptional repressor, activates ST18 expression in several liver cancer cells [75].

Although somatic L1 insertion likely plays a role in cancer development, few studies have directly demonstrated enhanced L1 retrotransposition during cancer development. Kaposi’s sarcoma-associated herpesvirus (KSHV), the causative agent of Kaposi’s sarcoma and primary effusion lymphoma, enhances L1 retrotransposition [77]. The inhibition of L1 reverse transcription suppresses KSHV-related tumorigenesis [77]. Capsaicin, an anti-tumor agent, suppresses L1 retrotransposition, suggesting that L1 retrotransposition plays a role in cancer development [78]. Naked mole-rats (NMR) show pronounced cancer resistance, and an L1 in the NMR genome exhibits extremely low retrotransposition activity, also suggesting that L1 retrotransposition plays a role in cancer development [79].

#### 2.2.2. L1 Expression

L1 5′ UTR harbors two internal promoters: sense and antisense promoters [36,39]. The sense promoter binds to RNA polymerase II and initiates L1 transcription [15]. L1 protein expression is a common feature of many high-grade malignant cancers [80]. The downregulation of L1 expression reduced cell proliferation in melanoma and prostate carcinoma cell lines [81]. L1 RNA and/or protein expression is critical for telomere maintenance in telomerase-positive tumor cells [82]. Transcription driven by the L1 antisense promoter (ASP) produces fusion transcripts of L1 and nearby genes [83]. The experimental activation of ASP activity enhances cell proliferation, suggesting that the hypomethylation of L1 observed in various cancers may stimulate cell proliferation through the activation of L1 ASP activity [34].

#### 2.2.3. L1 Chimeric Transcript

An ASP-driven chimeric transcript (*L1-MET*) between L1 and the receptor tyrosine kinase *MET* gene, which functions as an oncogene in many forms of cancer, has been observed in HCC (Figure 3B) [84,85]. MET activation initiates a complex network of biological responses that collectively induce invasive growth [86]. In HCC, the upregulation of the *L1-MET* transcript results in the accumulation of MET protein, which is associated with cancer metastasis and poor prognosis [84,85]. A chimeric transcript between L1 and *FGGY*, which encodes a protein that phosphorylates carbohydrates, is an L1 ASP-driven L1 chimeric transcript frequently observed in non-small cell lung cancer [35]. *L1-FGGY* initiates arachidonic acid metabolism reprogramming and activates the Wnt/β-catenin signaling pathway to promote tumor growth [35].

In some cases, the 3′ end processing machinery may bypass the L1 poly(A) signal and instead utilize a downstream poly(A) site [38], and, therefore, the sequence downstream of the 3′ end of L1s may be transcribed along with L1s. The resulting L1 chimeric transcript is reverse-transcribed and integrated into a new locus by the L1 retrotransposition machinery. This process is called 3′ transduction [15,37,38]. In a colorectal tumor, 35 somatic L1 retrotranspositions are identified, one of which has a 3′ transduction [87]. In another study, tumors from 53% of the 244 patients with cancer had somatic L1 retrotranspositions, of which 24% (655/2756 insertions) were 3′ transductions [88]. The transduction of the third exon of TPST1 has been identified in two primary lung cancers [88]. Thus, retrotransposition-competent L1s provide a vehicle to mobilize non-L1 sequences, such as exons or promoters, into existing genes [37]. The dispersion of exons and promoters can lead to the creation of new genes or alter the expression of existing genes [37]. The functional consequences of 3′ transductions in cancer are interesting areas for further investigation.

## 3. Involvement of HERVs, LINEs, and nrEVEs in Immunity

HERVs/LINEs and their products, including RNA, cytosolic cDNA, and proteins, are able to modulate and be influenced by the host immune system [24,89,90]. Recent evidence has shown links between HERVs and autoimmune diseases such as systemic lupus erythematosus, multiple sclerosis, and rheumatoid arthritis [23,91,92,93]. L1 dysregulation is becoming common in diseases with the chronic induction of type I interferon (IFN) signaling, such as Aicardi–Goutières syndrome (AGS) and Fanconi anemia (FA) [94,95]. HERVs/LTRs provide promoter/enhancer elements for immune-related gene expression as DNA elements, and HERV RNAs or proteins induce immune responses through innate immunity sensors [89,90,96]. Cytosolic L1 cDNA and L1 RNA are recognized by innate immunity sensors to trigger innate immunity as immunostimulatory DNA and RNA, respectively [94,95,97]. In addition to HERVs and LINEs, nrEVEs derived from ancient nonretroviral RNA and DNA viruses have been discovered in various mammalian genomes [2,3]. nrEVEs are possibly produced by reverse transcription and integration of viral mRNAs of ancient RNA viruses using the retrotransposon machinery [2]. It is speculated that nrEVEs, similar to HERVs and LINEs, play roles in antiviral immunity, as described below.

First, we briefly introduce the basic information regarding the innate immune system associated with HERVs and LINEs. When infection occurs, pathogen-associated molecular patterns (PAMPs), such as lipids, proteins, glycans, and nucleic acids, are recognized by innate immunity sensors, termed pattern recognition receptors (PRRs) [89,96,98,99]. HERV- and LINE-derived products are mainly recognized by three PRRs: toll-like receptors (TLRs), retinoic acid-inducible gene (RIG)-I-like receptors (RLRs), and cyclic GMP–AMP synthase (cGAS) [89,96]. TLRs are transmembrane PRRs that localize to the cell surface or endosomal compartments [96,100]. Upon binding to the corresponding PAMPs, TLRs dimerize and transduce signals to induce MyD88-dependent and TRIF-dependent pathways [100]. RLRs are a family of cytosolic RNA receptors that play key roles in the detection of viral RNA genomes and RNA replicative intermediates [89]. RIG-I and melanoma differentiation-associated protein 5 (MDA5) recognize distinct types of viral dsRNA ligands [101]. After binding to the viral RNA, RIG-I or MDA5 is recruited to the mitochondrial anti-viral signaling protein (MAVS) [98,102,103]. The interactions between RLRs and MAVS allow for downstream signal transduction. cGAS recognizes dsDNA derived from invading pathogens and induces an IFN response via the activation of the key downstream adaptor protein, the stimulator of interferon genes (STING) [104,105,106]. In addition, cGAS can interact with various types of nucleic acids, including DNA, DNA/RNA hybrids, and circular RNA, to contribute to a diverse set of biological functions, such as cellular senescence, antitumor immunity, and inflammation [104]. Following PRR stimulation, different downstream signaling pathways are activated, inducing an innate immune response through the transcription factors NF-κB and interferon regulatory factor 3/7 (IRF3/7). NF-κB stimulates proinflammatory cytokine expression and IRF3/7 induces type I IFN expression. IFNs are pro-inflammatory signaling molecules that stimulate the transcription of IFN-stimulated genes (ISGs), which are powerful restriction factors for infection [107,108,109].

### 3.1. HERVs and Innate Immunity

#### 3.1.1. LTRs as Promoter/Enhancer Elements

The innate immune pathway induces HERV expression [90]. HERV-K LTRs, which harbor two IFN-stimulated response elements (ISREs) activated by IFN signaling, lead to increased HERV-K expression in response to inflammation [110,111]. Vaccination also upregulates the expression of transcripts containing a HERV via innate immune activation [112].

**Figure 4:**
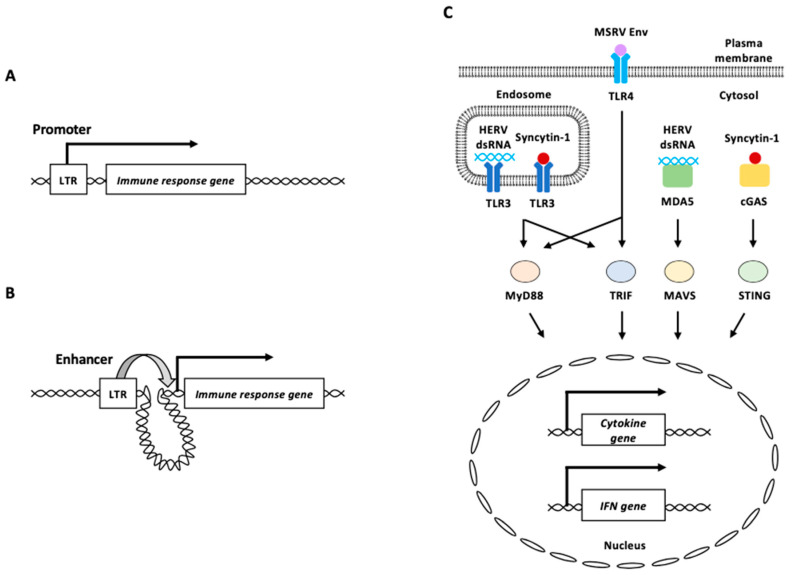
HERV functions in innate immunity. (**A**) HERV LTR functions as a promoter of an immune response gene. (**B**) HERV LTR functions as an enhancer of distant immune response gene from the element. (**C**) HERV-derived RNAs and proteins are recognized by PRRs. Endosomal TLR3 and cytosolic MDA5 recognize HERV dsRNA. Plasma membrane TLR4 potentially recognizes MSRV Env protein. Syncytin-1 is recognized by endosomal TLR3 or cytosolic cGAS. Recognition of HERV-derived RNAs and proteins by these PRRs activates signal transduction through adaptor proteins including MyD88, TRIF, MAVS, and STING, to induce innate immune responses, such as proinflammatory cytokine and/or IFN production. LTR: long terminal repeat, MSRV: multiple sclerosis-associated retroviral element, HERV: human endogenous retrovirus, IFN: interferon.

On the other hand, the binding sites of transcription factors observed on HERVs/LTRs are highly enriched in regions near the genes associated with innate immunity-related pathways such as “response to interferon-gamma” and “type I interferon signal pathway” (Figure 4A) [113]. The deletion of a subset of HERV elements in the human genome impairs the expression of adjacent IFN-induced genes such as *AIM2*, *APOL1*, *IFI6*, and *SECTM1* [114]. In addition, HERVs/LTRs in promoter-interacting regions appeared to function as transcriptional modulators of host genes via long-range interactions (Figure 4B) [113].

#### 3.1.2. HERV-Derived cDNAs, RNAs, and Proteins

HERV-derived RNAs and proteins are sensed mainly by three distinct types of the abovementioned PRRs [89,90]. HERVs can be expressed bidirectionally and produce dsRNA complexes owing to promoter activities of their flanking LTRs [115]. dsRNAs derived from aberrant HERV expression can act as dsRNA ligands for TLR3, a sensor of dsRNAs [116]. DNA methyltransferase inhibitors trigger the dsRNA formation and cause a type I IFN response and apoptosis in ovarian cancer [116]. The knockdown of the dsRNA sensors TLR3 and MAVS reduces this response.

γ radiation of HTP-1 human monocytes increases the expression of the HERV-K (HML-2) subfamily [117]. MDA5 and TLR3 bound to an equivalent number of copies of sense and antisense chains of HML-2 RNA was increased in γ-irradiated HTP-1 cells (Figure 4C) [117]. The binding of HML-2 RNA to MDA5 and TLR3 triggers MAVS-associated signaling pathways, resulting in the increased expression of type I IFN and inflammation-related genes [117].

Multiple sclerosis (MS)-associated retroviral element (MSRV) is an endogenous retrovirus belonging to the HERV-W family, and its expression correlates with that of the inflammatory cytokines, interleukin-6 (IL-6) and IL12p40, in patients with MS [89,118]. MSRV Env protein induces human monocytes to produce major proinflammatory cytokines [119]. Blocking experiments using neutralizing antibodies indicated that TLR4 and CD14 (a TLR4 co-receptor) were involved in the proinflammatory effects of MSRV Env on human monocytes [119]. TLR4 knockdown abolished the response to MSRV Env in a brain endothelial cell line, suggesting that MSRV Env is potentially recognized via TLR4 (Figure 4C) [120]. Several studies have reported abnormal increases in syncytin-1 protein levels in patients with schizophrenia, in whom increased innate immune activation and neuronal apoptosis are common [7,24,121,122,123,124,125]. The overexpression of syncytin-1 elevated the IL-6 levels in both human microglia and astrocytes [121]. TLR3 interacts with syncytin-1, and TLR3 deficiency impaired the expressions of IL-6 induced by syncytin-1, suggesting that TLR3 recognizes syncytin-1 to induce IL-6 expression (Figure 4C) [121]. Additionally, cGAS interacts with syncytin-1 and triggers IFN-β expression and neuronal apoptosis (Figure 4C) [124]. Syncytin-1 enhances cGAS and STING expression, as well as IRF3 phosphorylation in neuronal cells by repressing the expression of lncRNA01930 [124].

With aging, the loss of heterochromatin and the abnormal activation of HERVs can occur [126]. The upregulation of HERV-K-derived cDNA triggers the innate immune response, a part of the senescence-associated phenotype [127]. Extracellular HERV-K retrovirus-like particles can induce senescence, including the activation of innate immune responses, to non-senescent cells [127]. These results suggest that cDNAs and proteins derived from HERV-K might be associated with innate immune activation during aging [126].

While HERV RNAs and proteins can trigger innate immune responses as described above, some HERV-derived peptides have been implicated in immunosuppressive mechanisms [24,90]. Env transmembrane subunits have a characteristic immunosuppressive domain (ISD) that is conserved among retroviral Env proteins. The transmembrane subunit of the HERV-K sequence has been reported to inhibit T-cell activation [128]. The expression of HERV-H *env59* is negatively correlated with pathogenic factors, such as IL-6 and TLR7, in human autoimmune rheumatic diseases [129]. ISD of Env59 shows anti-inflammatory potential in a mouse model of arthritis [129]. The dimerized syncytin-2 ISD peptide induces ERK1 and ERK2 phosphorylation, which, in turn, inhibits cytokine production [130].

### 3.2. L1 and Innate Immunity

**Figure 5:**
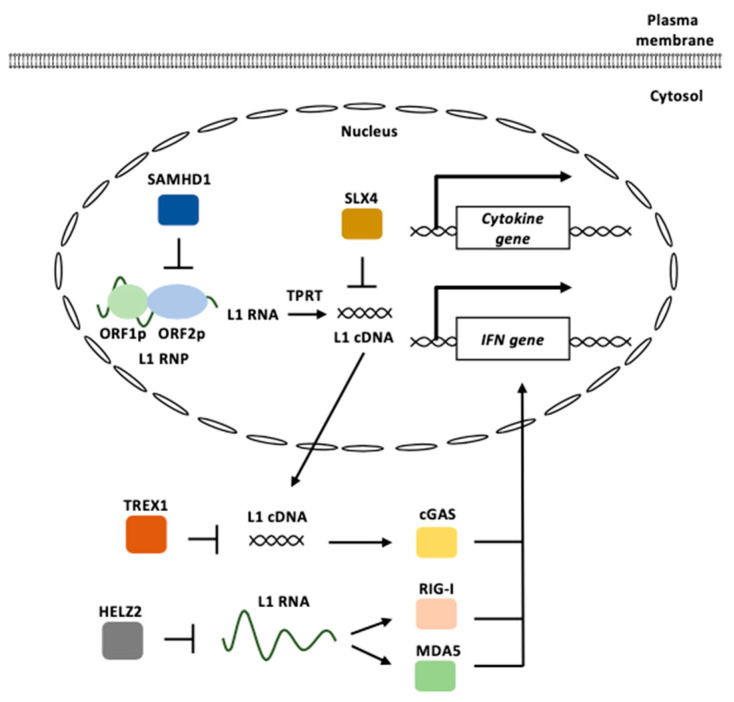
L1 functions in innate immunity. L1 RNPs are formed by ORF1p and ORF2p binding to an L1 RNA. cGAS recognizes cytosolic L1 cDNAs derived from the reverse transcription of the L1 RNA to induce type I IFN signaling. L1 RNAs are recognized by RIG-I and MDA5 to induce type I IFN signaling. TREX1 represses the accumulation of cytosolic L1 cDNAs. HELZ2 reduces L1 RNA levels. SAMHD1 inhibits ORF2p-mediated L1 reverse transcription. SLX4 prevents the accumulation of reverse-transcribed L1 cDNA. L1 RNP: L1 ribonucleoprotein, TPRT: target-primed reverse transcription, IFN: interferon.

L1 contributes to autoimmunity through cGAS-, RIG-I-, and MDA5-mediated DNA/RNA sensing pathways (Figure 5) [94,95,97]. The accumulation of cytosolic L1 cDNAs and RNAs is closely associated with dysregulated innate immunity, as described in this section. For example, TREX1 is an anti-viral DNase that prevents the accumulation of cytosolic DNAs and the subsequent type I IFN-associated inflammatory response [131,132]. TREX1-deficient neural precursor cells, neurons, and astrocytes exhibit an increase in intracellular DNA species, which correlates with neuronal toxicity [133]. L1 is a major source of accumulated DNA in TREX1-deficient neural cells [133]. Mutations in *TREX1* have been found in patients with AGS, a disease that perturbs the innate immunity [134]. The inhibition of L1 reverse transcription leads to a reduction in extrachromosomal DNAs and rescues the associated neurotoxicity. Mutations in the *SAMHD1* gene, which encodes a deoxynucleoside triphosphate triphosphohydrolase (dNTPase), have also been observed in patients with AGS [135,136,137]. SAMHD1 acts as a potent cellular restriction factor against retroviruses, such as HIV and simian immunodeficiency virus, through its dNTPase activity [138,139]. However, the enzymatic active site mutant of SAMHD1 maintains substantial anti-L1 activity [140]. Instead, SAMHD1 inhibits ORF2p-mediated L1 reverse transcription in L1 RNPs by reducing ORF2p levels [140].

*SLX4* encodes a protein that functions as an assembly component of multiple structure-specific endonucleases [141]. The SLX4 endonuclease complexes are required to repair specific types of DNA lesions in the nucleus [142]. The absence of SLX4 causes cytosolic DNA accumulation, including sequences derived from active L1, which triggers the cGAS-STING pathway to elicit IFN expression [143]. Mutations in *SLX4* have been found in patients with FA, a genetic disorder characterized by elevated cancer susceptibility and proinflammatory cytokine production [144]. The treatment of FA cells with a reverse transcriptase inhibitor decreased the accumulation of cytosolic DNAs and pro-inflammatory signaling.

Helicase with zinc finger 2 (HELZ2), which exhibits exoribonuclease activity, is associated with L1 ORF1p [145]. HELZ2 recognizes RNA sequences and/or RNA structures within the L1 5′ UTR and reduces L1 RNA. The overexpression of HELZ2 reduces the L1 RNA to induce IFN-α expression [145]. The activation of L1 retrotransposons increases the expression of IFN and IFN-stimulated genes and IFN suppresses the replication of L1 [146].

### 3.3. Non-Retroviral Endogenous Viral Elements and Innate Immunity

nrEVEs derived from the nucleoprotein (N) gene of bornaviruses, EBLNs, are present in the genomes of several mammalian species, including humans [147]. *Homo sapiens* EBLN-1 (hsEBLN-1) RNA potentially affects the expression of a neighboring *COMMD3* gene [148,149]. Since COMMD proteins interact with different NF-κB subunits, hsEBLN-1 RNA may regulate immune responses through the COMMD3-NF-kB pathway [148]. Several EBLNs give rise to abundant PIWI-interacting RNAs (piRNAs) in the male gonad that are antisense to the current bornavirus N mRNA [150]. Although it needs to be proved, this finding raises the possibility that EBLN-derived piRNAs may exhibit anti-bornavirus activity. An EBLN element in the genome of the thirteen-lined ground squirrel (*Ictidomys tridecemlineatus*), itEBLN, encodes an ORF with 77% amino acid sequence identity to the current bornavirus N protein [151]. itEBLN co-localizes with the viral factory in the nucleus and appears to affect bornavirus polymerase activity by being incorporated into the viral RNP [151].

Endogenous filovirus-like elements (EFLs) are another nrEVE found in the mammalian genomes [152,153,154,155]. A filovirus VP35-like element (mlEFL35)-derived protein (mlEFL35p) contains nearly full-length amino acid sequences corresponding to Ebola virus VP35 [156]. Ebola virus VP35 binds to dsRNA, leading to the inhibition of type I IFN production [157]. The expression of mlEFL35p also inhibits human IFN-β promoter activity, suggesting that mlEFL35p potentially acts as an IFN antagonist [156].

## 4. Conclusions and Perspectives

In this review, we summarize current knowledge regarding the physiological roles of HERVs and LINEs in cancer development and innate immunity. Dysregulated HERV/LINE expression is closely associated with cancer and immunological diseases. HERVs/LTRs serve as regulatory elements for the expression of genes with oncogenic potential and those in the host immune regulatory network. Several HERV-derived RNAs and proteins act as oncogenic molecules. HERV-derived RNAs and proteins are generally recognized as PAMPs by PRRs, such as TLRs, RLRs, and cGAS, and activate downstream signaling pathways, whereas some HERVs have immunosuppressive activity. Understanding the mechanisms determining how and when the host activates or suppresses the immune system via HERV modulation is important. L1 insertions within or near an oncogene or a tumor suppressor gene are found in many types of cancers. The functional consequences of L1 insertions and 3′ transductions in cancer are also interesting areas for further investigation. Although very little is known about the biological impact of L1 chimeric transcripts, some of them have been shown to activate various oncogenic pathways. The aberrant accumulation of cytosolic L1 cDNA is found in autoimmune diseases such as AGC and FA. Consistently, accumulated cytosolic L1 cDNAs and L1 RNAs are recognized by PRRs and induce IFN signaling pathways. Finally, although the functions of nrEVEs are not fully known, they are highly likely to play roles in various biological processes including immune system.

In summary, many studies have suggested that HERVs and LINEs play various roles in cancer development and immune responses. However, we are still far from understanding the full functions of HERVs and LINEs. The functions of HERVs and LINEs described in this review are the tip of the iceberg. Further studies are needed to reveal the roles of HERVs/LINEs in various biological processes, such as neuronal development, apoptosis, and stress responses. Additionally, further studies are required to understand the role of nrEVEs in antiviral immunity and other biological pathways. As environmental factors affect the expression of HERVs/LINEs [27], it is interesting to clarify the multifaceted functions of HERVs/LINEs from the perspective of genetic and environmental factors.

## Data Availability

Data are contained within the article.
